# Comparative analysis of external and internal loads in preparation male volleyball and beach volleyball matches

**DOI:** 10.1186/s13102-025-01302-3

**Published:** 2025-10-14

**Authors:** Bruno Figueira, Artūr Vincėlovič, Nuno Batalha, Rūtenis Paulauskas

**Affiliations:** 1 Departamento de Desporto e Saúde, Escola de Saúde e Desenvolvimento Humano, Universidade de Évora, Largo dos Colegiais 2, Évora, Portugal; 2 Comprehensive Health Research Centre (CHRC), Universidade de Évora, Évora, Portugal; 3 Educational Research Institute, Education Academy, Vytautas Magnus University, Kaunas, Lithuania

**Keywords:** Tracking technologies, Intensity zones, Physiological demands, Internal load, External load

## Abstract

**Background:**

This study analyzed external and internal load demands in preparation volleyball and beach volleyball matches.

**Method:**

Twelve national-level male players (age = 21.9 ± 2.9 years, height = 188.7 ± 7.7 cm, body mass 83.7 ± 7.7 kg) participated in three beach volleyball and one indoor volleyball sessions. External loads—including total distance covered, movement speed zones, high-intensity accelerations and decelerations, and jump counts by height—were assessed using VXSport (Omni) inertial units.

**Results:**

External loads showed no significant differences except for higher jump counts (< 20 cm) in beach volleyball. However, beach volleyball elicited greater physiological responses, including higher average and peak heart rates, increased time in the 90–100% heart rate maximum zone, and elevated energy consumption.

**Conclusion:**

These findings emphasize the impact of environmental constraints, such as sand surfaces, on amplifying internal workloads. The results highlight the need for tailored training programs addressing the specific demands of each volleyball format. Additionally, this research provides benchmarks for designing targeted exercises, improving real-time monitoring, and enhancing sport-specific conditioning strategies. By exploring biomechanical and physiological distinctions between volleyball and beach volleyball, the study contributes to optimizing athlete performance and guiding sports training methodologies.

## Background

Monitoring internal (IL) and external load (EL) is essential for designing training aligned with match demands [1]. Volleyball and beach volleyball involve intermittent efforts, with EL quantified by duration, distance covered, velocity, acceleration, and jump metrics [2]. IL reflects physiological strain, assessed via heart rate (HR), blood lactate (BLa), and perceived exertion [3]. Understanding IL–EL dynamics informs training to optimize performance and prevent overload [4, 5]. However, data on male volleyball players remain scarce, particularly studies combining local positioning system (LPS), inertial sensors, and HR monitors for comprehensive load assessment [6, 7].

Seasonal transitions between indoor and beach volleyball serve competitive and conditioning purposes. Beach volleyball introduces distinct demands due to sand instability, environmental exposure (e.g., wind, sun), and larger relative court area [5, 8]. These factors alter physical and technical requirements, necessitating adaptation. Evidence suggests that 12 weeks of structured beach training enhances lower-limb endurance and vertical jump in indoor players [8]. Additionally, elite female beach athletes cover greater distances and face higher physical loads during rallies than indoor counterparts [9]. However, data on the specific demands during transitions from sand to court remain limited.

Various thresholds have been proposed to classify locomotor actions (e.g., walking, sprinting, acceleration) in team sports [10–12]. However, tracking-based data for volleyball remain scarce. Volleyball involves high-intensity, skill-specific actions—particularly vertical jumps, which are central to performance (e.g., spike, block, serve) [13–15]. Studies show that elite Austrian players achieve higher spike jumps on hard courts (67.7 ± 5.7 cm) than on sand (60 ± 2.7 cm) [16]. Unlike other sports, volleyball lacks standardized zones for categorizing jump intensity. Proposed thresholds in similar contexts define jumps as low (< 20 cm), medium (20–40 cm), or high (> 40 cm) intensity [17–19]. However, how jump intensities differ between sand and hard surfaces remains unknown.

Heart rate-based IL monitoring is a cornerstone of performance analysis in team sports [19–21]. The Summated Heart Rate Zones (SHRZ) model by Edwards [22] is widely applied across disciplines such as basketball and rugby [23], offering valid estimates of physiological load by classifying HR data into five intensity zones based on %HRmax. These zones correlate well with EL, reinforcing SHRZ as a reliable marker of training and match demands [22]. While volleyball and beach volleyball share locomotor patterns, differences in surface, team size, and court dimensions significantly affect load profiles [1, 23]. Yet, little is known about how these variables shape movement speed, jump intensity, and HR distribution—especially in male athletes. Thus, understanding these sport-specific demands is essential for optimizing training and promoting skill transfer. To address these gaps, the present study aims to systematically compare EL and IL experienced by male athletes during preparation volleyball and beach volleyball matches.

## Materials and methods

### Study design

A crossover design trial was used to assess EL and IL during preparation volleyball and beach volleyball matches. Twelve well-trained male volleyball players performed three beach volleyball and one volleyball match. Several EL and IL variables were assessed.

### Participants

Twelve (*n* = 12) national-level male volleyball players participated in this study (age = 21.9 ± 2.9 years, height = 188.7 ± 7.7 cm, body mass 83.7 ± 7.7 kg). From October to March, the athletes trained and competed regularly in indoor volleyball, practicing five times per week and playing one match weekly in national competitions. From May to September, the players participated in Baltic and national beach volleyball tournaments organized by the National Volleyball Federation. On average, they had 9.0 ± 4.6 years of experience in indoor volleyball and 5.0 ± 1.5 years in beach volleyball. Before the study began, all participants were in good health and free of injuries. Each athlete provided written informed consent. The study protocol was approved by the local Institutional Research Ethics Committee (SA-EK-24-53) and complied with the ethical guidelines outlined in the Declaration of Helsinki.

### Procedure

This study investigated two types of volleyball formats by analyzing four preparation matches: three beach volleyball (BVOL) sessions and one indoor volleyball (VOL) session. These matches were conducted under standardized competitive-like conditions—mimicking official gameplay in terms of rules, intensity, and structure—thus categorized as preparation matches designed to replicate real-match demands without the presence of formal competition. The VOL teams were organized based on participants’ skill levels and playing positions to ensure balanced competition. During the BVOL sessions, each VOL team was divided into six smaller teams, each comprising three positional pairs: setter/ middle blocker, outside hitter /libero and opposite/outside hitter. These teams competed against opponents matched by similar positional compositions. Adhering to established methodologies [24, 25], participants engaged in 30 min of BVOL and VOL, simulating one set of match-play in each format. To maintain consistent physical conditions, all BVOL matches were conducted on the same day, while the VOL match was scheduled on a separate day. Environmental conditions were carefully standardized. The BVOL matches took place outdoors on International Volleyball Federation (Fédération Internationale de Volleyball (FIVB))-compliant sand courts (8 m wide, 16 m long, with a net height of 2.43 m) under controlled conditions: cloudy skies, an air temperature of 19 °C, wind speeds of 2 m/s, and relative humidity of 68%. The VOL match was played indoors on a standard wooden volleyball court (9 m wide, 18 m long, with a net height of 2.43 m), where the air temperature was maintained at 20 °C and the relative humidity at 61%. Environmental conditions, including temperature, relative humidity, and wind speed, were continuously monitored during all match simulations using a portable weather station (e.g., Kestrel 5500, Nielsen-Kellerman, USA). Measurements were taken at baseline and at 15-minute intervals throughout each match to ensure consistency across sessions. Before each match, participants completed a standardized 20-minute warm-up under the supervision of a strength and conditioning coach. The warm-up included dynamic stretching and volleyball-specific movement exercises designed to optimize performance and reduce injury risk. All matches were conducted in accordance with official FIVB rules and officiated by a certified referee. To ensure the continuity of play and simulate realistic match-play, coaches retrieved balls between points in adherence to standard match protocols, removing time-outs and side-switches.

### Data collection and processing

#### External-load data collection

EL was operationalized using a set of mechanical and locomotor indicators commonly validated in intermittent team sports, including total distance covered, high-speed running, speed zone distribution, accelerations, decelerations, and jump height categories. These variables, recorded through wearable inertial units (VXSport Omni), are recognized proxies for biomechanical loading and have been adopted in recent EL frameworks [6, 11]. The VXSport (Omni) athlete-monitoring system was employed to measure EL variables using inertial measurement devices (VXSport, Wellington, New Zealand), which sampled data at 100 Hz [26]. The devices were positioned securely between the scapulae using a manufacturer-designed vest to ensure stability during the preparation BVOL and VOL matches. These triaxial devices, equipped with gyroscopes, accelerometers, and magnetometers, captured movement data across all planes. Calibration of the units was performed as a one-time procedure, tailored for each player to align the device’s tracking capabilities with their gait for indoor VOL testing. This calibration occurred seven days before the preparation matches. The process required players to visit a standard athletic track equipped with a global positioning system (GPS) signal, ensuring minimal interference from tall buildings or dense foliage. Players completed a series of activities, including walking, jogging, striding, and sprinting at maximal effort over 125 m. The calibration process followed a detailed protocol provided by VX Sport technicians (https://support.vxsport.com/hc/en-us/articles/4407258662543-VX-Omni-Calibration). According to Smith et al. [26], the VXSport Omni system demonstrated high to nearly perfect reliability for indoor applications, with intraclass correlation coefficients ranging from 0.77 to 0.99 for the selected indicators. Key performance metrics included High-Speed Running (HSR, measured as the number of instances > 15.00 km·h⁻¹), High-Intensity Accelerations (ACC), High-Intensity Decelerations (DEC > 3 m·s − 2), and Total Distance Covered (TDC, in meters). To evaluate movement intensities, speed zones were defined based on the categories established by Núñez-Sánchez et al. [27]. Distances traveled within each speed zone were classified as follows: SZ 1 (0.00–7.00 km·h⁻¹), SZ 2 (7.01–13.00 km·h⁻¹), SZ 3 (13.01–18.00 km·h⁻¹), and SZ 4 (> 18.01 km·h⁻¹). In addition to these metrics, EL data included Jump Rate (JR, measured in jumps/min) and jump counts categorized by height zones (HZ), as defined by Fox et al. [17]. Jump heights were classified into three zones: HZ1 (< 20 cm), HZ2 (20–40 cm), and HZ3 (> 40 cm).

#### Internal-load data collection

VXSport devices were employed to analyze continuous HR measurements, using Suunto HR sensors (Suunto Smart Sensor, Suunto Oy, Finland) [26]. These sensors were integrated into a manufacturer-designed vest that facilitated strapless HR monitoring. The HR devices were positioned between the scapulae, adjacent to the VXSport inertial measurement devices. The vest, equipped with integrated HR sensors, enabled seamless data collection during match-play. HR data were transmitted to the VXSport devices via Bluetooth and used to calculate average HR (HRavg, bpm), peak HR (HRpeak, bpm), and energy consumption (EC, in calories). The SHRZ model proposed by Edwards [28] was applied to quantify IL across five HR zones, each spanning 10% of HRmax. These zones were defined as follows: zone 1: 50–59.9% HRmax, zone 2: 60–69.9% HRmax, zone 3: 70–79.9% HRmax, zone 4: 80–89.9% HRmax, zone 5: 90–100% HRmax. HRmax was calculated for each participant using the age-predicted equation: HRmax = 208 – (0.7 × age) [21, 29]. After data collection, EL and IL data were downloaded, processed, and stored using VXSport software (VXSport release 7.1.0.4). The processed data were subsequently exported for further analysis. To evaluate the intensity of anaerobic-glycolytic metabolism, BLa concentration (mmol/L) was measured three minutes after completing the preparation BVOL and VOL matches. Blood lactate samples were collected from participants’ fingertips and analyzed immediately using a validated lactate analyzer (Lactate Pro, Arkray, Tokyo, Japan).

### Statistical analysis

A descriptive analysis was conducted, providing means and standard deviations for the dataset. Normality assumptions of the data were assessed using the Shapiro-Wilk test. To compare BVOL and VOL, paired t-tests were applied for performance-related variables that followed a normal distribution. For variables that did not meet the normality criteria, the Wilcoxon test was employed. Complementarily, effect sizes (ES) for the variables following a normal distribution were analysed according to Cohen’s d using the following thresholds: small (0.2); medium (0.5); and large (0.8); while the variables that did not show a normal distribution, the ES was calculated by subtracting the average values and the division of the result by the combined standard deviation converted to the following r values: small (0.10); medium (0.30); and (0.50) (large) [30]. The alpha level for all statistical tests was set a priori at α = 0.05 and calculations were carried out using SPSS software (IBM Corp. Released 2016. IBM SPSS Statistics for Windows, Version 24.0. Armonk, NY: IBM Corp).

## Results

No statistically significant differences (*p* > 0.05) were found between volleyball and beach volleyball matches for any of the external load parameters analyzed, including total distance covered, player load, number of accelerations and decelerations (Table 1).

**Table 1:** Comparison of locomotor external load metrics between beach volleyball and indoor volleyball players

Variables	BVOL	VOL	t	*p*	95% CI for Cohens’s d		
					Cohen’s d	Lower	Upper
TDC (m)	1351.50 *±* 201.61	1343.33 *±* 204.58	−0.098	0.922	−0.039	−0.840	0.760
Distance SZ 1 (0.00–7.00 km·h⁻¹) (m)	794.83 *±* 93.04	781.08 *±* 67.57	−0.414	0.683	−0.163	−0.969	0.634
Distance SZ 2 (7.01–13.00 km·h⁻¹) (m)	452.17 *±* 151.69	449.08 *±* 126.39	−0.054	0.957	−0.021	−0.822	0.778
Distance SZ 3 (13.01–18.00 km·h⁻¹) (m)	92.50 *±* 28.59	93.83 *±* 57.71	0.072	0.943	0.028	−0.771	0.829
Distance SZ 4 (> 18.01 km·h⁻¹) (m)	12.00 *±* 10.74	19.33 *±* 18.24	1.200	0.243	0.473	−0.328	1.298
HSR (num)	31.83 *±* 9.54	30.92 *±* 15.74	−0.173	0.865	−0.068	−0.870	0.731
ACC (num)	51.42 *±* 11.33	45.08 *±* 19.18	−0.985	0.335	−0.388	−1.206	0.411
DEC (num)	16.42 *±* 5.63	17.33 *±* 7.48	0.339	0.738	0.134	−0.664	0.938

Notes: BVOL = Beach Volleyball; VOL = Volleyball; TDC = Total distance covered; SZ = Sprint zone; km = kilometers; h = hour; m = meters; HSR = High-Speed Running; ACC = Acceleration; DEC = deceleration

Except for low-height jumps in the HZ1 category (< 20 cm), which were significantly less frequent in beach volleyball players compared to indoor volleyball (*p* = 0.018, ES = − 1.007), both groups exhibited comparable performance across all other jump-related variables (Table 2).

**Table 2:** Comparison of jump-related external load metrics between beach volleyball and indoor volleyball players

Variables	BVOL	VOL	t	*p*	95% CI for Cohens’s d		
					Cohen’s d	Lower	Upper
JR (Jump/min)	1.42 *±* 0.67	1.17 *±* 0.58	−0.980	0.338	−0.386	−1.205	0.413
JC HZ1 (< 20 cm), (num)	10.25 *±* 5.48	5.33 *±* 3.80	−2.555	0.018*	−1.007	−1.889	−0.176
JC HZ2 (20–40 cm), (num)	18.75 *±* 11.89	11.92 *±* 8.66	−1.610	0.122	−0.634	−1.473	0.173
JC HZ3 (> 40), (num)	17.17 *±* 10.96	13.83 *±* 10.55	−0.759	0.456	−0.299	−1.112	0.499
Avg Jump HZ1 (< 20 cm), (cm)	14.27 *±* 0.90	15.00 *±* 1.35	1.552	0.135	0.612	−0.195	1.448
Avg Jump HZ2 (20–40 cm), (num)	27.50 *±* 2.20	26.75 *±* 3.39	−0.644	0.526	−0.254	−1.064	0.544
Avg Jump HZ3 (> 40), (num)	46.08 *±* 4.70	49.17 *±* 4.76	1.596	0.125	0.629	−0.178	1.467

Notes: BVOL = Beach Volleyball; VOL = Volleyball; JR = jump rate; JC = jump count; HZ = height zone; cm = centimeters; num = number; Avg = average

Significant differences between beach volleyball and indoor volleyball players were observed in several internal load variables: beach volleyball players demonstrated lower average heart rate (HRavg; *p* < 0.001, ES = − 1.877), lower peak heart rate (HRpeak; *p* = 0.01, ES = − 1.106), and reduced time spent in zone 5 (90–100% HRmax; *p* = 0.015, ES = − 1.044), while showing greater time in zone 3 (70–79.9% HRmax; *p* = 0.009, ES = 1.122) and a lower training load score (C; *p* < 0.001, ES = − 1.767) (Table 3).

**Table 3:** Comparison of internal load variables between beach volleyball and volleyball players

Variables	BVOL	VOL	t	*p*	95% CI for Cohens’s d		
					Cohen’s d	Lower	Upper
HR_avg_ (bpm)	166.67 *±* 11.99	143.25 *±* 11.97	−4.788	< 0.001*	−1.877	−2.925	−0.955
HR_peak_ (bpm)	185.67 *±* 8.16	173.25 *±* 12.98	−2.806	0.01*	−1.106	−2.002	−0.266
HRZ1 (50–59.9% HRmax), (min)	0.0 *±* 0.0	0.0 *±* 0.0	-	-	-	-	-
HRZ2 (60–69.9% HRmax), (min)	0.0 *±* 0.0	0.0 *±* 0.0	-	-	-	-	-
HRZ3 (70–79.9% HRmax), (min)	0.44 *±* 1.08	4.65 *±* 5.01	2.848	0.009*	1.122	0.281	2.021
HRZ4 (80–89.9% HRmax), (min)	22.96 *±* 5.70	23.87 *±* 4.27	0.441	0.663	0.174	−0.624	0.980
HRZ5 (90–100% HRmax), (min)	6.60 *±* 6.10	1.48 *±* 2.76	−2.649	0.015*	−1.044	−1.932	−0.210
EC (cal)	238.83 *±* 21.04	197.17 *±* 24.37	−4.483	< 0.001*	−1.767	−2.780	−0.852
Bla (mmol/L)	2.73 *±* 0.88	2.97 *±* 0.73	0.704	0.489	0.278	−0.520	1.089

Notes: BVOL = Beach Volleyball; VOL = Volleyball; HR = heart rate; avg = average; bpm = beats per minute; Z = zone; max = maximum; min = minute; EC = energy consumption; cal = calories; Bla = blood lactate; mmol = millimoles; L = liters

## Discussion

The present study aims to systematically compare external and internal load experienced by male athletes during preparation volleyball and beach volleyball matches. BVOL elicits a greater number of vertical jumps within the < 20 cm height range, reflecting higher neuromuscular demands. It is also associated with elevated average and peak heart rates (HR_avg_ and HR_peak_), indicating increased cardiovascular strain. Players in BVOL spend significantly more time in HRZ1 (90–100% HR_max_) and less time in HRZ3 (70–79.9% HR_max_), highlighting differences in intensity distribution. Additionally, BVOL results in higher calorie expenditure, further emphasizing its greater overall physiological load compared to VOL.

The EL profile of volleyball includes horizontal actions such as passing, setting, and digging, as well as vertical jumps during attacks, blocks, and serves [31]. These actions generate both mechanical and neuromuscular strain and are central to performance monitoring and training prescription. In our study, TDC—a core EL metric—was comparable between BVOL and VOL athletes, despite structural differences in playing surfaces and game dynamics. This finding contrasts with team sports where larger playing areas typically yield greater TDC [32, 33], suggesting that the high frequency of short, explosive movements and brief rally durations in volleyball (~ 6s rally, ~ 14s rest) [34, 35] constrain locomotor volume independently of court size. The average TDC observed (1343.33 ± 204.58 m) exceeds values reported in prior literature for professional male players in 3-set matches (1221 ± 327 m) [36], potentially due to contextual differences (e.g., tactical strategies, measurement tools, match intensity). Speed zone analysis revealed no significant differences in locomotor velocity structure between BVOL and VOL, indicating that, despite surface-related constraints, movement intensity is relatively stable. Similarly, comparable distributions of HSR, ACC, and DEC suggest matched EL between formats. However, the higher variability in HSR and ACC in VOL aligns with Mroczek et al.’s [36] conclusions on role-specific locomotor profiles, particularly in specialized court positions.

The vertical jump is a cornerstone of volleyball performance, critical to executing effective spikes, blocks, and jump serves [37]. In the present study, JC in HZ1: <20 cm was significantly greater in BVOL compared to VOL, suggesting a higher frequency of submaximal jumps in the sand-based format. This may reflect adaptations to surface constraints, where reduced ground reaction forces limit vertical displacement and necessitate repeated lower-intensity actions. However, no significant differences were observed between BVOL and VOL in HZ2 or HZ3, nor in average jump height or JR, indicating similar neuromuscular demands in maximal effort jump execution across formats. These results provide valuable context to the EL profile of each sport. Previous studies have shown that jump distribution varies by format and tactical context. For example, Vilamitjana et al. [38] reported that in VOL, jumps predominantly occur during blocking (37.9%), attacking (21.7%), and serving (17.6%), whereas in BVOL, Turpin et al. [39] observed a higher proportion of smash (44%) and block jumps (39%). The increased JC in BVOL’s HZ1 likely reflects a higher repetition of preparatory and reactive jumps on sand, where instability imposes greater mechanical demand during propulsion and landing phases [40, 41]. Our findings align with the notion that while peak jump performance may be conserved between formats, the distribution of jump loads—particularly at lower intensities—differs due to biomechanical constraints of the sand surface. This is supported by the lack of differences in HZ2 and HZ3, suggesting that when athletes do engage in high-intensity jumps, the resultant load is comparable. Moreover, as in previous work by Stankovic et al. [42] positional variability (e.g., liberos performing 95% of jumps during setting) likely contributes to inter-player differences in jump load. Hence, monitoring JC by intensity zone offers a refined understanding of movement-specific EL and informs targeted conditioning strategies tailored to surface and position-specific demands.

Despite similar EL metrics across match conditions, our results reveal significantly higher heart rate responses in BVOL, as reflected by elevated HR_avg_ and HR_peak_ compared to VOL. Specifically, BVOL elicited HR_avg_ of 165 ± 20 bpm and HR_peak_ of 188 ± 6 bpm, consistent with findings in beach soccer [43] yet notably exceeding values reported for beach handball [44]. These data highlight the intensified cardiovascular demands of BVOL, likely driven by the unstable sand surface and greater locomotor cost associated with horizontal and vertical displacement on sand [45–47]. To further elucidate IL profiles, we examined the distribution of time spent in SHRZ. Although both formats presented the highest effort accumulation within HRZ4 (80–89.9% HRmax), a pronounced difference was observed in HRZ5 (90–100% HRmax), with BVOL athletes sustaining this intensity for 6.60 ± 6.10 min versus only 1.48 ± 2.76 min in VOL. This divergence underscores a greater reliance on high-intensity cardiovascular output in BVOL, aligning with the significantly greater EC recorded in this condition. These findings position HRZ distribution and EC as key IL parameters characterizing sport-specific physiological strain.

BLa levels showed no significant differences between both conditions, suggesting that glycolytic energy production played a minimal role in either scenario. Previous research has documented mean BLa levels of 2.30 ± 0.46 mmol/L for BVOL [48] and 2.7 ± 1.2 mmol/L for VOL [49]. These findings closely align with the values observed in our study, underscoring the consistency of metabolic responses across different volleyball formats, and reinforcing the reliability of these measurements in evaluating energy system contributions.

## Conclusion

This study investigated EL and IL during male preparation matches of VOL and BVOL. The analysis revealed no significant differences in EL, except for JC in HZ1 (< 20 cm). However, BVOL elicited greater physiological responses than VOL, including higher HR_avg_, HR_peak_ during matches, more time spent in the 90–100% HR_max_ zone, and increased energy consumption. These findings highlight the impact of environmental constraints, such as the sand court surface, in significantly increasing internal workloads. This emphasizes the necessity of developing tailored training programs to meet the distinct demands of each volleyball format. Moreover, the results provide valuable benchmarks for designing targeted exercises and implementing real-time monitoring strategies, offering practical applications for coaches, sport scientists, and performance analysts.

## Limitations

This study presents several limitations that should be considered when interpreting the findings. First, participants had greater experience in VOL than in BVOL, which may have influenced their performance and physiological responses. Second, the simulation match utilized a small sample size, restricting the ability to analyze positional differences. Since player positions serve distinct roles on the court, they are likely to demonstrate variations in EL and IL characteristics. Finally, psychophysiological stress in real matches is expected to exceed that of preparation matches, potentially impacting the generalizability of these results to competitive settings.

## Data Availability

Data will be made available upon reasonable request.
